# A Neonate with Autosomal Dominant Pseudohypoaldosteronism Type 1 Due to a Novel Microdeletion of the *NR3C2* Gene at 4q31.23

**DOI:** 10.3390/children8121090

**Published:** 2021-11-25

**Authors:** Su Jin Kim, Dasom Park, Woori Jang, Juyoung Lee

**Affiliations:** 1 Department of Pediatrics, Inha University Hospital, Incheon 22332, Korea; kimsjped@inha.ac.kr (S.J.K.); ekthadl2564@naver.com (D.P.); 2 Department of Pediatrics, Inha University College of Medicine, Incheon 22332, Korea; 3 Northwest Gyeonggi Regional Center for Rare Disease, Inha University Hospital, Incheon 22332, Korea; jangwr@inha.ac.kr; 4 Department of Laboratory Medicine, Inha University College of Medicine, Incheon 22332, Korea

**Keywords:** pseudohypoaldosteronism, mineralocorticoid receptors, *NR3C2* gene, dehydration, hyponatremia, hyperkalemia, neonate

## Abstract

Dehydration with hyponatremia can occur from a variety of causes and can be potentially fatal to infants. Pseudohypoaldosteronism type 1 (PHA1) is a rare disease that can cause severe dehydration along with hyponatremia and hyperkalemia because of renal tubular unresponsiveness to mineralocorticoids. Autosomal dominant PHA1 (ADPHA1, OMIM #177735) is caused by inactivating mutations in the *NR3C2* gene, which encodes the mineralocorticoid receptor, and it can lead to renal salt-wasting, dehydration, and failure to thrive during infancy. Here, we report a case of a 20-day-old female neonate who presented as severe dehydration with hyponatremia and polyuria. We suspected that her diagnosis might be PHA1 based on markedly elevated plasma renin activity and serum aldosterone levels. For the genetic diagnosis of PHA1, we performed targeted exome sequencing of all causative genes of PHA1, but the result was negative. We confirmed by chromosomal microarray that a novel heterozygous microdeletion was found in the 4q31.23 region spanning exons 7–9 of the *NR3C2* gene, and the patient was diagnosed with ADPHA1. In conclusion, our patient is a case of ADPHA1 that developed into a salt-wasting crisis in the neonatal period due to a microdeletion of the 4q31.23 region inherited from her father.

## 1. Introduction

Dehydration with hyponatremia can occur in infants for a variety of causes, including impairments in oral intake, viral gastroenteritis, use of diuretics, and heart or renal diseases. The most frequent cause of life-threatening hyponatremia associated with hyperkalemia in newborns and infants is congenital adrenal hyperplasia (CAH). Pseudohypoaldosteronism type 1 (PHA1) is a rare disease with an estimated prevalence of 1 per 80,000 newborns. PHA1 can cause severe dehydration with hyponatremia and hypokalemia due to resistance of aldosterone, it might be fatal to neonates and infants. [1,2]. Two different genetic forms of PHA1 have been defined as follows: (1) renal type with autosomal dominant PHA1 (ADPHA1, OMIM #177735) caused by mutations in the *NR3C2* gene encoding mineralocorticoid receptor [3,4] and (2) systemic type with autosomal recessive PHA1 (ARPHA1, OMIM #264350) caused by mutations of any of the three genes (*SCNN1A, SCNN1B*, and *SCNN1G*) encoding the ENaC channel. As ENaC is expressed not only in the distal tubules but also in the sweat glands, salivary glands, colon, and lungs, excessive salt-wasting occurs in these organs. Therefore, patients with ARPHA1 show more severe symptoms and have an earlier onset [5,6,7]. In PHA1, resistance to mineralocorticoid action results in sodium wasting and impairment in potassium and hydrogen secretion in the distal nephron, which leads to hyponatremia, hyperkalemia and metabolic acidosis despite elevated plasma renin activity and aldosterone levels [3,4].

Urgent correction of dehydration and electrolyte imbalance is important since severe dehydration with hyponatremia and hyperkalemia are potentially fatal to infants. Although initial management should be universal with the correction of water loss and treatment of electrolyte imbalance, glucocorticoid therapy is not as effective in restoring salt and water balance in PHA1 as in the case of CAH. To overcome the underlying resistance to aldosterone, PHA1 patients require high-dose sodium chloride supplementation [1,2,3,4].

Here, we report a case of a 20-day-old female neonate who presented as severe dehydration with hyponatremia and polyuria and was later diagnosed with ADPHA1 with a microdeletion in chromosome 4q31.23, which spans exons 7–9 of the *NR3C2* gene.

## 2. Case Presentation

A 20-day-old girl was admitted to the neonatal intensive care unit with a chief complaint of poor oral intake through the emergency room. She was lethargic and did not suck well with swallowing only 10 to 20 mL of formula at a time in the last two days. However, the amount of urine did not decrease, and diapers were changed 10 to 14 times per day. Vomiting and diarrhea were not observed. She was born at 38^+2^ weeks of gestation with 3380 g (50th–75th percentile) via cesarean section. No abnormal findings were noted during the prenatal and immediate postnatal periods. She was the first child of healthy, nonconsanguineous Korean parents, and her family history was unremarkable. At admission, her weight was 3100 g (25th–50th percentile), length was 53 cm (50th–75th percentile), and head circumference was 36 cm (50th–75th percentile). Although vital signs were appropriate for her age (heart rate 150 beats/min, blood pressure 78/50 mmHg, respiratory rate 48 breaths/min, and body temperature 36.5 °C), her lips were dry, and the capillary refill time was prolonged to 5–6 s. Physical examination revealed both thumbs in palms, frontal bossing, prominent upper lip, high arched palate, sparse frontal scalp hair, and bilateral 5th finger clinodactyly. An initial capillary blood gas analysis showed severe metabolic acidosis (pH 7.16, pCO_2_ 28.3 mmHg, pO_2_ 42 mmHg, HCO_3_^−^—17.3 mmol/L, base excess—17.3 mmol/L). With an impression of dehydration, 20 mL/kg normal saline was infused intravenously for over 1 h before other laboratory results were obtained.

**Figure 1:**
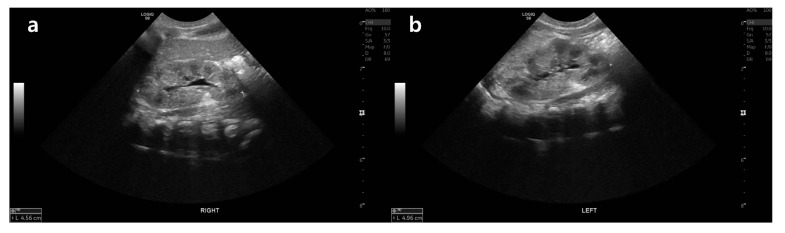
Renal ultrasonography of the patient. (**a**) Mild hydronephrosis with grade 1 pelvic dilatation in the right kidney. (**b**) No abnormal findings in the left kidney.

The laboratory tests at admission were as follows: serum sodium 113.3 mEq/L, serum potassium 8.79 mEq/L, serum chloride 90.8 mEq/L, total CO_2_ 8.1 mEq/L, serum lactic acid 1.0 mmol/L, serum ketone body 24 µmol/L, blood glucose level 83 mg/dL, blood urea nitrogen 55.1 mg/dL, and serum creatinine 0.65 mg/dL. Her urinalysis revealed a specific gravity of 1.014 and pH 5.0 and was negative for white blood cells and red blood cells. Her spot urine sodium and potassium levels were 74 and 27.7 mEq/L, respectively. The serum and urine osmolality values were 232 and 229 mOsm/kg, respectively. All the results of the neonatal screening test were normal, which included TSH (1.2 mIU/L), 17-hydroxyprogesterone (1.6 ng/mL), total galactose (1.0 mg/dL), and mass spectrometry for amino acid, organic acid, fatty acid, purine, peroxisome, and carbohydrate metabolic disorders. The plasma ammonia level was within the normal limit as 97 µg/dL. The plasma renin activity and serum aldosterone level were markedly elevated to 142.0 ng/mL/h (normal range, 0.32–1.84 ng/mL/h) and 4560 ng/dL (normal range, 4.2–20.9 ng/dL), respectively. Renal ultrasonography revealed no abnormalities except mild hydronephrosis in the right kidney (Figure 1). No abnormal findings were found in cardiac echocardiography or brain magnetic resonance imaging.

To correct severe hyponatremia, 60 mL of 3% sodium chloride was initially intravenously administered over 8 h. Her urine output on the first day of admission was 8.45 mL/kg/h. Hyponatremia and hyperkalemia were improved with intravenous fluid and oral sodium chloride supplementation (8 mEq/kg/day). The patient consumed an adequate amount of milk (170–200 mL/kg/day), and weight gain was appropriate (40–80 g/day) after oral sodium chloride supplementation.

**Figure 2:**
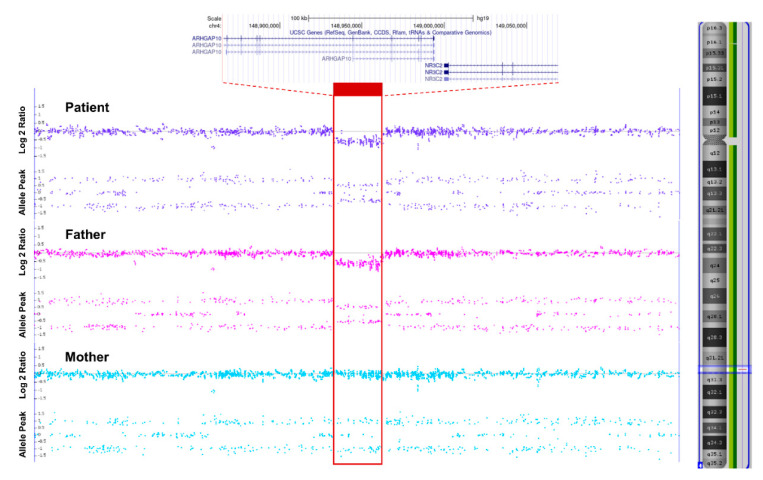
Schematic of a deletion spanning exons 7–9 in our patient and her parents (as shown in the red bar).

For the genetic diagnosis of the patient, targeted exome sequencing (TES) was performed. Genomic DNA was extracted from proband blood. All exon regions of all human genes (~22,000) were captured by a Twist Human Core Exome Kit (Twist Bioscience, South San Francisco, CA, USA). The captured regions of the genome were sequenced using a NovaSeq 6000 sequencing machine (Illumina, San Diego, CA, USA). In TES, no other pathogenic/likely pathogenic single-nucleotide variants (SNVs) or small insertion and deletion variants associated with the clinical phenotypes were identified. However, her clinical phenotypes and biochemical results indicated PHA1. Therefore, we performed a chromosomal microarray (CMA) to identify deletion-encompassing genes responsible for PHA1. CMA (CytoScan Dx, Affymetrix Cytogenetics, Santa Clara, CA, USA) revealed a 203 kb heterozygous deletion at 4q31.23: arr[GRCh37] 4q31.23(148865586_149069090)x1 (Figure 2). This deletion spans exons 7–9 of *NR3C2* and exons 15–23 of the *ARHGAP10* gene. Haploinsufficiency of the *NR3C2* gene, which encodes the mineralocorticoid receptor, is responsible for ADPHA1. However, the details of the functional role of the *ARHGAP10* gene in human disease remain unclear. Parental testing showed that the deletion was paternally inherited. Her father had no history of clinical PHA1 manifestation and had normal plasma electrolytes and serum aldosterone values with only slightly elevated plasma renin activity at testing.

## 3. Discussion

Our patient presented with severe dehydration with hyponatremia, hyperkalemia, and metabolic acidosis. Although she had severe dehydration, her urine amount had not been reduced. We suspected PHA1 as her diagnosis because the 17-hydroxyprogesterone level was normal. This clinical diagnosis was supported by markedly elevated plasma renin activity and serum aldosterone levels. PHA1 is a rare disease, but it may be underdiagnosed because of its variable clinical courses, including asymptomatic cases. Owing to the rarity of the disease, the diagnosis of PHA1 is difficult in the clinical setting. Clinical suspicion through careful physical examination and analysis of electrolyte levels, urinalysis, and hormonal evaluation, including plasma renin activity and aldosterone levels, is important.

ADPHA1 (OMIM #177735) is caused by inactivating mutations in the *NR3C2* gene, which encodes the mineralocorticoid receptor and was first reported by Geller et al. in 1998 [3]. It is characterized by isolated renal resistance to mineralocorticoids, leading to renal salt-wasting, dehydration, and failure to thrive during infancy [4,8,9]. Although most patients with ADPHA1 show a milder clinical course than ARPHA1, it has been reported to be associated with a high infant mortality rate [4]. Since the causative gene for ADPHA1 was identified, more than fifty different SNVs have been reported [10]. However, there are few reports of ADPHA1 due to copy number variants (CNVs), including deletion or inversion of the 4q31.23 region where the *NR3C2* gene is located [11,12,13,14,15].

In our case, clinical and laboratory findings suggest PHA1, and physical examination showed mild dysmorphic features. Therefore, TES was performed for genetic diagnosis, but causative genetic variant was not found. However, a few cases in which PHA1 was diagnosed by CNV in the 4q31.23 region containing the *NR3C2* gene have been reported, and TES has lower sensitivity to CNVs detection than CMA, so CMA was performed. The results of CMA revealed a novel microdeletion in the 4q31.23 region, confirming that this microdeletion spanned exons 7–9 of the *NR3C2* gene. Thus, the patient was diagnosed ADPHA1.

Since the genetic causes of PHA1 involve at least four genes, targeted NGS panels that include all of these genes can be considered the first diagnostic option to be implemented. As in this case, if PHA1 is strongly suspected clinically but is not diagnosed by NGS or direct sequencing, it is thus necessary to confirm with CMA or qMPSF.

Recently, Zennaro et al. reported that approximately 62% of genetically confirmed *NR3C2* gene mutations are inherited from one parent [10]. In addition, Hanukoglu et al. reported in a family study spanning 40 years that even families with the same mutation had different clinical manifestations and renin/aldosterone levels [14]. The causes of various clinical phenotypes in individuals or families with identical mutations in the *NR3C2* gene are still not well understood. Several other factors may be involved in distal sodium reabsorption, such as infection, prematurity, and the action of other unknown genes. Even in this case, the father of the patient with the same deletion did not present with the clinical features of PHA1 in the past medical history, and there were no significant abnormalities in plasma electrolytes, renin activity, or serum aldosterone value. The natural course of ADPHA1 shows a tendency toward the alleviation of symptoms and a decrease in renin/aldosterone levels with age [14]. Therefore, prevention of salt-wasting crisis and growth restriction in infancy through oral NaCl supplementation with early diagnosis through genetic testing is important for patient prognosis.

In conclusion, our patient is a PHA1 case that developed as a salt-wasting crisis in the neonatal period due to a novel microdeletion of the 4q31.23 region that includes the *NR3C2* gene. This will help to broaden the genetic and clinical spectra for 4q31.23 deletions associated with ADPHA1.

## Data Availability

All data and material analyzed in this study are included in this published article.
